# In vivo MRI volumetric measurement of prostate regression and growth in mice

**DOI:** 10.1186/1471-2490-7-12

**Published:** 2007-07-24

**Authors:** Kent L Nastiuk, Hui Liu, Mark Hamamura, L Tugan Muftuler, Orhan Nalcioglu, John J Krolewski

**Affiliations:** 1 Department of Pathology and Laboratory Medicine, University of California, Irvine, CA, USA; 2 Center for Functional Onco-Imaging, University of California, Irvine, CA, USA; 3 Chao Family Comprehensive Cancer Center, University of California, Irvine, CA, USA

## Abstract

Background

Mouse models for treatment of late-stage prostate cancer are valuable tools, but assessing the extent of growth of the prostate and particularly its regression due to therapeutic intervention or castration is difficult due to the location, small size and interdigitated anatomy of the prostate gland *in situ*. Temporal monitoring of mouse prostate regression requires multiple animals and examination of histological sections.

**Methods:**

Initially, T2-weighted magnetic resonance imaging (MRI) was performed on normal year-old C57/BL6 mice. Individual mice were repeatedly imaged using inhalation anesthesia to establish the reproducibility of the method and to follow hormone manipulation of the prostate volume. Subsequently, MRI fat signal was suppressed using a chemical shift-selective (CHESS) pulse to avoid signal contamination and enhance discrimination of the prostate.

**Results:**

High field (7T) MRI provides high resolution (117 × 117 μm in plane), highly reproducible images of the normal mouse prostate. Despite long imaging times, animals can be imaged repeatedly to establish reliability of volume measurements. Prostate volume declines following castration and subsequently returns to normal with androgen administration in the same animal. CHESS imaging allowed discrimination of both the margins of the prostate and the dorsal-lateral lobes of the prostate (DLP) from the ventral lobes (VP). Castration results in a 40% reduction in the volume of the DLP and a 75% reduction in the volume of the VP.

**Conclusion:**

MRI assessment of the volume of the mouse prostate is precise and reproducible. MRI improves volumetric determination of the extent of regression and monitoring of the same mouse over time during the course of treatment is possible. Since assessing groups of animals at each time point is avoided, this improves the accuracy of the measurement of any manipulation effect and reduces the number of animals required.

## Background

Androgens regulate the growth of both normal and neoplastic prostate. Thus, for almost fifty years the principal treatment for advanced stage prostate cancer has been androgen ablation therapy by orchiectomy or, more recently, pharmacological treatment with anti-androgens or gonadotropin inhibitors. While most such prostate cancers initially regress, they eventually become independent of androgens for growth and metastasize fatally. Mouse models of prostate cancer may prove very useful in dissecting the signaling pathways important for both progression to cancer and inhibition of tumor cell growth [1]. As in humans, androgens promote mitosis and differentiation of rodent prostate ductal epithelium, and further, appear to inhibit apoptosis of differentiated cells [2]. After androgen ablation, the balance between mitosis and apoptosis is disrupted, and the prostate undergoes a wave of cell death, involuting the gland [3]. However, in mice, assessing prostate regression due to therapeutic intervention or castration is difficult due to the intra-abdominal location, small size and interdigitated anatomy of the gland [4].

MRI of mouse prostate allows longitudinal assessment of volume changes in an individual prostate over time. To maximize utility, MRI should have very high resolution, since the mouse prostate is only 20 mm^3^, and should allow successive monitoring of the same mouse. Xu et al. [5] successfully imaged a cohort of xenograft-bearing mice on two separate occasions using a commercial 1.5T instrument, but the resulting resolution was only 0.39 mm^3^. Hsu et al [6] followed tumor development in TRAMP mice with up to four imaging sessions in a 7T MRI with a resolution of 0.175 mm^3^. Other investigators imaged the TRAMP mouse prostate *in situ *on a single occasion at higher resolutions [7]. The effect of chemopreventive drugs on the volume of the TRAMP mouse prostate was assessed at a single post-manipulation time point by MRI [8,9]. The effect of castration on tumor volume in TRAMP mice was monitored in the same mouse up to four times over many months [10] but again the resolution was only 0.175 mm^3^. Instead of assessing tumor volume, a single 2-D image was used to assess cross-sectional area of prostates developing cancer in the absence of the tumor suppressor genes PTEN and p53 [11]. Fricke, et al. [12] recently reported using a 7T MRI 3-D imaging procedure to measure prostate volume in normal and knock-out animals at a high resolution (0.005 mm^3^) and suggest that longitudinal studies would be possible.

Our goals in this study are to improve the reproducibility, precision, and resolution of mouse prostate volume determination by MRI. Additionally, by developing a technique to allow survival of the mouse, and hence imaging the same animal over time during hormone level manipulations, we will be able to greatly reduce the number of animals needed to precisely determine mouse prostate volume changes.

## Methods

### Animals

All procedures were approved by the UC Irvine committee on animal care. Male C57/BL6 mice (retired breeders more than 1 yr old) were purchased from the NCI or Charles River Labs. Mice were housed individually in the UCI animal facility and all procedures were in accordance with institutional guidelines. Animals were anesthetized with Xylazine (13 mg/kg) and Ketamine (87 mg/kg), and castrated via scrotal incision. Each testicle, vas deferens and accompanying fat pad were removed and the blood vessels and vas deferens ligated. The incision was closed with surgical silk rather than wound clips for compatibility with MRI. Androgen was replaced using 5 mg/kg 5alpha-androstan-17beta-ol-3-one (dihydrotestosterone, DHT, Sigma) in corn oil injected sub-cutaneously daily. There was neither weight gain nor loss greater than 10% for any animal during these manipulations. For imaging, mice were anesthetized as above, and positioned inside the instrument. Anesthesia was maintained using 1% isoflurane, 99% oxygen delivered via a nose cone for the duration of the imaging.

### MRI

Animals were placed on a removable stage, insulated for warmth and held in position by wrapping in paper towel and plastic wrap. They were then positioned inside a shielded 8-leg highpass birdcage coil (65 mm diameter), constructed in-house, which was used for RF pulse transmission and signal reception. Experiments were performed on a MRRS (MR Research Systems Ltd., Surrey, UK) console interfaced to a 7T Oxford 300/150 horizontal bore magnet with an inner bore size of 150 mm (Oxford Instruments Limited. UK). The system is equipped with an actively shielded gradient coil (Model BFG-150/90-S, Resonance Research Inc, Billerica, MA) with a maximum gradient strength of 750 mT/m, 100 μs rise time and inner diameter of 90 mm. The gradients are driven by Techron series 7700 linear gradient amplifiers, with a maximum slew rate of 150 T/m/s.

In pilot studies, T1- and T2-weighted images were used to determine the optimal parameters for imaging the mouse prostate *in vivo *(data not shown, DNS). T2-weighted images were superior due to less fat interference. The echo time of the T2-weighted images also affected image quality, with 20 msec differentiating prostate best from the surrounding tissue (DNS). Maintaining anesthesia using injectables (Xylazine/Ketamine) proved difficult due to decreased respiration and body temperature, and so a combination of initial anesthesia with injectables Ketamine/Xylazine and maintenance anesthesia using isoflurane/oxygen inhalation inside the magnet gave 90–100% survival, depending on the imaging session duration. Longer total anesthesia time increased the incidence of respiratory failure and death, which we need to avoid in order to image the same animal over multiple sessions. Using the final, optimized anesthesia paradigm, we were able to collect data by averaging 8 image sets which gave an adequate image signal to noise ratio and which allowed limiting the imaging time to 2.5 h (3.5 h including initial imaging to position the mouse and shim the magnet) resulting in 100% survival from the anesthesia and hypothermia due to the magnet. We successfully imaged the same mouse up to 3 times over a period of 14 days to assess normal prostate volume determination reproducibility, and subsequently imaged the same mouse up to 8 times over a period of 40 days (b7m1) and 10 times over 37 days (b5m2) to follow castration-induced regression and DHT-induced re-growth of the prostate. Finally, images were collected from two test mice and the identification of each organ on the MRI was confirmed by gross examination (DNS). A total of 21 mice were used in these studies. Four mice were not imaged, eight mice were imaged on a single occasion, while nine mice were imaged on multiple occasions.

For our initial studies, scout proton spin-echo images in the axial orientation (TR/TE = 1080 ms/23 ms, 25 slices, slice thickness = 0.5 mm, image matrix size = 128 × 128 FOV = 35 mm^2^), were used to accurately localize the position of the mouse prostate. Then a 2-D interleaved multi-echo spin-echo sequence (TR/TE = 4500 msec/20 ms, 32 slices, slice thickness = 0.3 mm with no separation, image matrix size = 256 × 256, field of view (FOV) = 30 × 30 mm, NEX = 8 averages) was used to produce images for volume determination.

Subsequently, imaging was performed with a chemical suppression sequence (CHESS) to repress the signal from the abdominal fat. After scout images were obtained, spectroscopy identified the resonant frequency of the lipid-derived protons (typically 950–1000 Hz). A Gaussian 240 Hz pulse was used for CHESS fat suppression using the lipid resonance frequency and a pulse duration of 1000 μs. A 2-D interleaved CHESS spin-echo with fat suppression sequence (TR/TE = 4500 msec/25 ms, 32 slices, slice thickness = 0.2 mm with no separation, image matrix size = 256 × 256, FOV = 30 × 30 mm, NEX = 8 averages) was used to acquire the fat-suppressed images of mouse prostate.

### Volume determination

**Figure 1:**
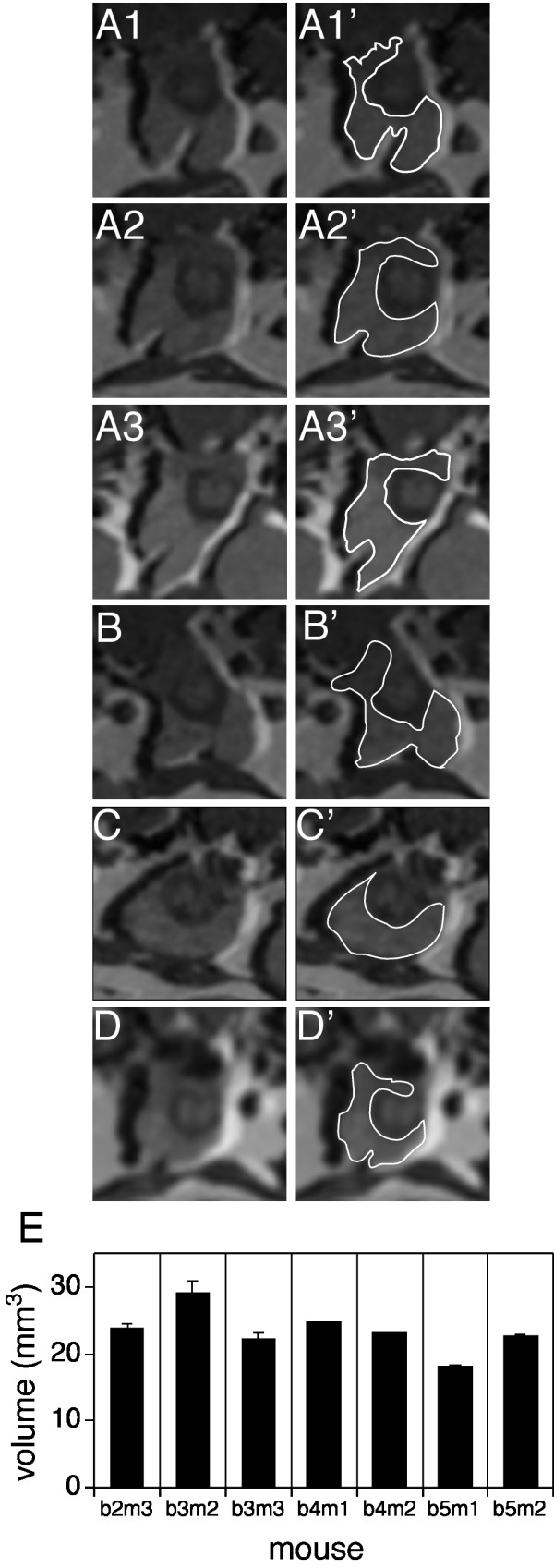
MRI of normal mouse prostate. Prostates from year old C57/BL6 mice were imaged (FOV = 35 mm^2^, 256 × 256 pixels). **A**: Images of b3m3 on three different days (**A1**, **A2**, **A3**). **B**: b3m2, **C**: b4m1, **D**: b2m3. Second column (**A1'**-**D'**) shows the prostate outlined in white. Each image is the central 8.75 mm square (64 × 64 pixels) portion of the complete MRI image. **F**: Plot of prostate volumes for the indicated mice, in cubic millimeters. Error bars represent standard error of the mean of the prostate volume for each animal that was imaged multiple times (b2m3, b3m2, and b3m3 were imaged 3 times, b5m1 and b5m2 were imaged twice and b4m1 and b4m2 were imaged once).

Each series of 32 image slices was analyzed using NIH ImageJ [13]. These were assembled as a stack and the circumference of the whole prostate was outlined on each slice (typically 10–20 slices total) containing identifiable prostate (illustrated in Figure 1, panels A1', A2' and A3'), and the number of bounded pixels in each slice was computed. The number of pixels in each bounded slice was added and multiplied by the voxel size, 0.0041 mm^3^, to yield the prostate volume. Voxel size is the volume of one pixel in the MRI. It is the image resolution, 117 × 117 μm (computed by dividing the FOV, 30 mm^2^, by the number of points in the image matrix, 256 × 256) and multiplied by the slice thickness. The voxel size for the initial (non-CHESS) studies was 0.0041 mm^3^, and for the subsequent CHESS images it is 0.0027 mm^3^. Volumes were determined by a single segmenter at least twice for each image session and agreed within 5%, or they were redetermined. For the initial studies, multiple images were captured of most mice before treatment (normals), and the mean volumes and accompanying standard error of the means are presented. In the reproducibility study, the coefficient(s) of variation (CV) was calculated as the appropriate SD divided by the mean and expressed as a percentage.

## Results

### Imaging of the normal prostate

Prostate volumes were measured in mature mice. Figure 1 (Panels A1–A3) shows prostate image slices from one mouse (batch 3, mouse 3 (b3m3)) obtained on three separate days over an eight day span. Panels A1–A3 show the slice with the largest cross-sectional area of the prostate from each session. The circumference of the whole prostate was outlined on each slice (typically 10–20 slices total) containing identifiable prostate (illustrated in Figure 1, Panels A1'-A3'). The number of pixels in each bounded slice was added and multiplied by the voxel size, 0.0041 mm^3^, to yield the prostate volume (22.14 +/- 1.02 mm^3 ^(mean +/- SE, n = 3) for mouse b3m3). Other normal mature mice show similar prostate size (Figure 1, panels B', C', and D'). The average volume from 7 individual mature mouse prostates, is 23.80 +/- 2.62 mm^3 ^(mean +/- SD, n = 7) in year old mice, where the individual values are illustrated in Figure 1E. To assess the reproducibility of the measurements over multiple imaging sessions, the coefficient of variation was determined for individual animals to range from 1.7% to 10.9%, and overall was 6.5% for the five mice imaged multiple times (80% CI upper bound = 13.6%). We find the volume of the whole prostate varies between individual mice, but that one imaging session is sufficient to determine baseline prostate volume for the subsequent studies.

### Imaging regression of the prostate following castration

**Figure 2:**
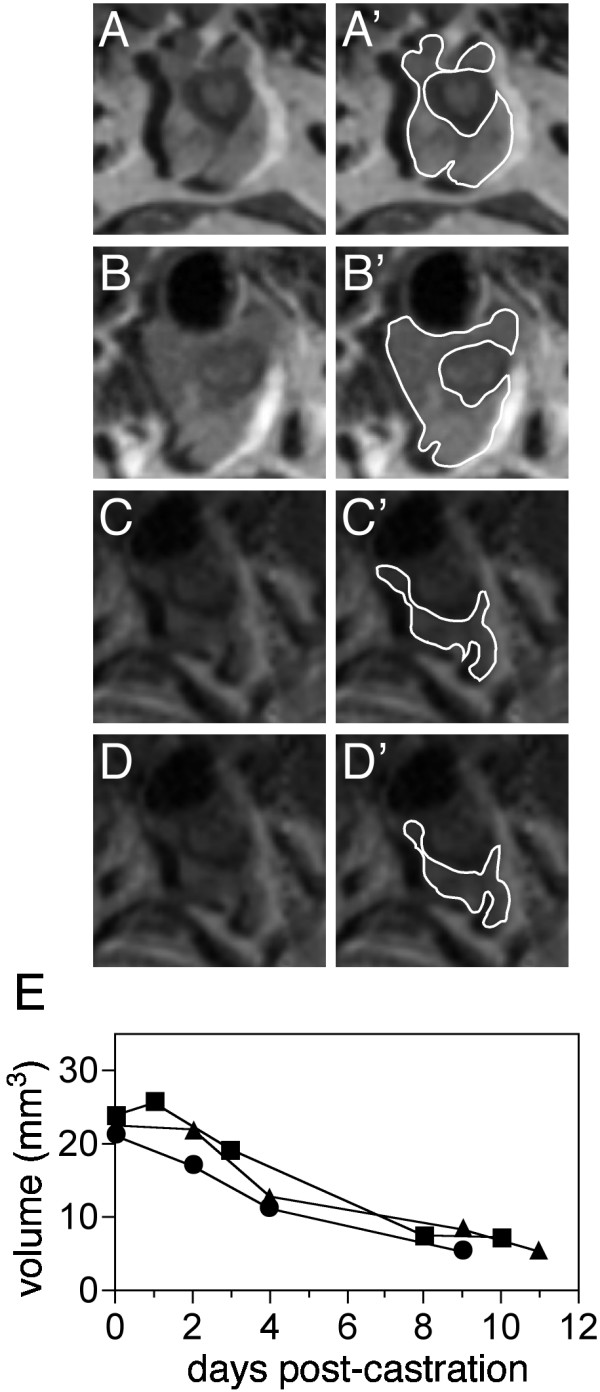
MRI of prostate following castration. Mouse b2m3. **A**: 1 day; **B**: 3 days; **C**: 8 days; and **D**: 10 days. The second column (**A'**-**D'**) shows the prostate outlined in white as in Figure 1. **E**: Regression of the prostates of three individual mice. Square symbols represent the volume of the prostate of b2m3, circles represent b5m1, and triangles represent b5m2.

Three mice from Figure 1 were castrated following MRI assessment of normal prostate volume, and reduction of the prostate volume was assessed by MRI over 11 days. Figure 2 shows the prostate image of maximum cross-sectional area for imaging sessions performed on mouse b2m3 one (A), three (B), eight (C), and ten (D) days after castration. The total volume of the prostates from three separate mice declined ~75% following castration (Figure 2E), to 6.04 mm^3 ^+/- 1.05 mm^3 ^(mean +/- SD, n = 3).

Figure 2 also illustrates two problems. Fat surrounding the prostate gland produces an intense signal which appears to 'shift' into the prostate and, as the prostate regresses, its composition (of water, fat and prostatic fluid, or other constituents) changes over time, reducing the signal to noise ratio, thus hampering our ability to differentiate the prostate from surrounding tissues in the MR images.

### Optimizing MRI by CHESS reveals prostate lobes

**Figure 3:**
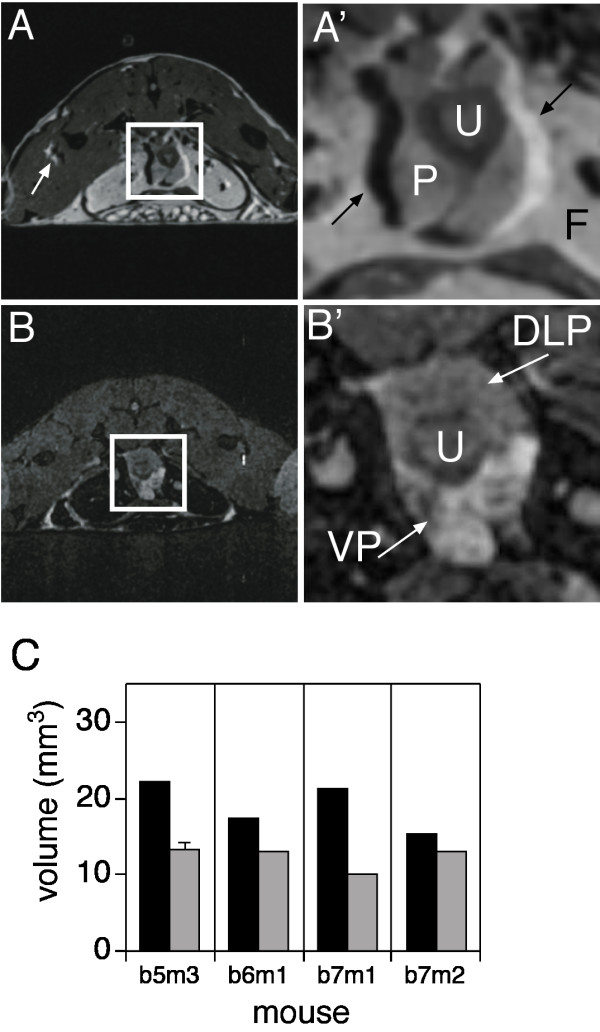
CHESS suppresses fat signal artifact and facilitates discrimination between ventral and dorsal-lateral lobes. **A: **T2- weighted MRI. **A'**: Magnification of the central 64 × 64 pixel square, indicated by the white box in **A**. White arrow in A and black arrows in **A' **illustrate the shift of the signal derived from fat. The locations of the prostate (P), ureter (U) and abdominal fat (F) are indicated. **B**: T2-weighted image acquired using CHESS. **B'**: Magnification as in **A'**. White arrows indicate ventral prostate (VP) and dorsal-lateral prostate (DLP). **C**: Volume of the VP (solid bars) and DLP (grey bars) of normal mice determined using CHESS images, as in Figure 1F.

The MRI signal shift due to fat is illustrated in Figure 3. The white arrow in panel A indicates a region of fat signal shift of several millimeters, within the muscle. The intense fat signal shift, down and to the left, leaves a dark area, up and to the right, corresponding to the region vacated by the shifting fat signal. In the magnified image (panel A') of the central portion of Figure 3, the left black arrow points to a void resulting from the fat signal shift, and the right arrow to a hyperintense region where the fat signal has shifted into the prostate. This fat signal complicates the drawing of the border of the prostate as some estimation of the underlying margin is required.

Fat signal can be greatly reduced by selectively suppressing fat protons prior to acquiring the water proton signal, using a chemical shift selective imaging sequence (CHESS). The frequency of fat proton emission is determined during pilot imaging, and a pre-saturation pulse is applied prior to slice image acquisition. Figure 3B shows an image slice of normal prostate acquired using CHESS. The intense abdominal fat (labeled F in Figure 3A') and other fat signals have been eliminated, and the prostate borders are well delineated (Figure 3B'), greatly facilitating precise quantitation. An unexpected but very valuable result of the CHESS imaging is the greater signal intensity in the ventral lobe relative to the dorsal-lateral region (Figure 3B', VP versus DLP). This may be due to higher the water content of the prostatic fluid in the more numerous secretory ducts of the ventral lobe.

We used CHESS imaging to determine the volume of normal mouse prostate lobes. At the same time, we were able to increase the accuracy of our volumetric determinations by reducing the slice thickness to 200 μm, resulting in a voxel size of 0.0027 mm^3^. The solid bars in Figure 3C show the volumes of the VPs derived from four normal mice (solid bars; 19.04 +/- 2.78 mm^3^, mean +/- SD, n = 4). The grey bars show the DLPs, whose average volume is 12.39 +/- 1.29 mm^3 ^(mean +/- SD, n = 4) from four normal mice. Although Figure 1 demonstrates good reproducibility and inter-animal agreement for total prostate volume, comparison with the CHESS images shows that standard T2 MRI underestimates total (VP + DLP) prostate volume (23.80 versus 31.43 mm^3^).

### Regression and re-growth of individual lobes of the mouse prostate

**Figure 4:**
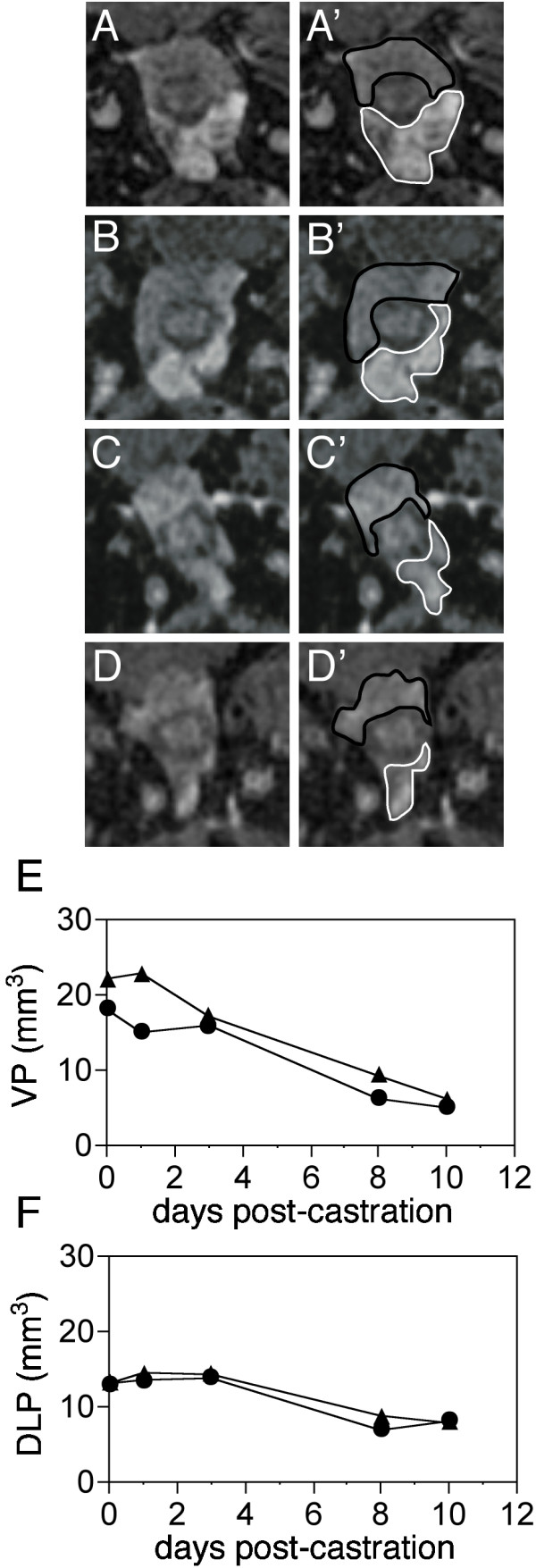
MR-CHESS imaging of prostate involution. Prostate of b6m1 following castration **A**: 1 day; **B**: 3 days; **C**: 8 days; **D**: 10 days. Second column (**A'**-**D'**) shows the VP outlined in white and the DLP outlined in black. Each image is the central 7.5 mm square (64 × 64 pixels) portion of the complete MRI image (FOV = 30 mm^2^, 256 × 256 pixels). **E**: Plot of volume during regression of the VPs of two individual mice. **F**: Plot of volume during regression of the DLPs of two individual mice. Triangle symbols represent the volume of the prostate of b5m3 and circles represent the volume of b6m1.

The ventral lobe of the mouse prostate regresses more than the dorsal or lateral lobes (which are difficult to distinguish from each other in the mouse). Figure 4 shows a time course (similar to Figure 2) of mouse prostate CHESS images following castration (panels A-D). Panels A'-D' show the same slices with the ventral prostate outlined in white and the dorsal-lateral in black. CHESS imaging greatly enhances the signal to noise ratio in the images at the later time-points and the elimination of the fat signal overlap allows more accurate delineation of the lobe boundaries. The volume of the ventral prostate is reduced 71.67 +/- 0.07% (mean +/- SD, n = 3) over 10 days (Figure 4E), while the the dorsal-lateral prostate regresses only 33.78 +/- 8.43% (mean +/- SD, n = 3, Figure 4F). Thus, MRI demonstrates that prostate lobe-specific reductions in mouse are similar to rat.

**Figure 5:**
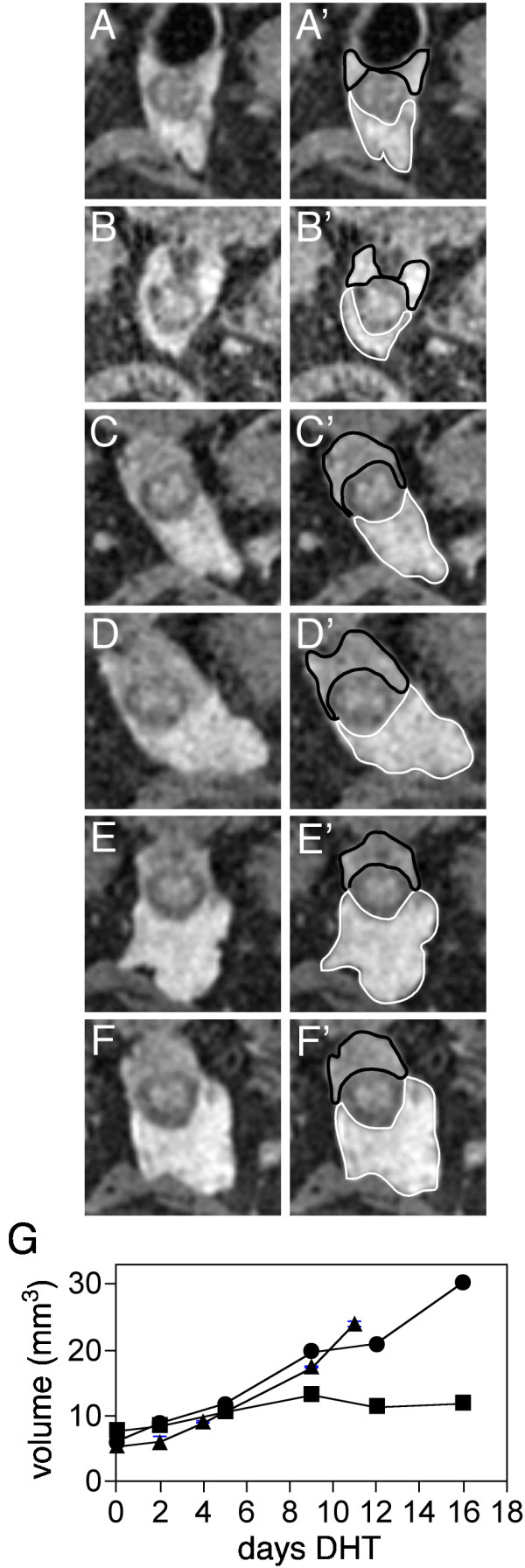
MR-CHESS imaging of prostate re-growth during androgen supplementation of castrated mice. Prostate from b7m1 following castration and DHT treatment. **A**: day 0 (16 days following castration); **B**: 2 days DHT treatment; **C**: 4 days DHT treatment; **D**: 9 days DHT treatment; **E**: 12 days DHT treatment; **F**: 16 days DHT treatment. Second column (**A'**-**F'**) as in Figure 4. **G**: Plot of prostate volume during re-growth of the ventral prostates of two individual mice. Triangle symbols represent the volume of the prostate of b5m2, circles represent the volume of the VP of b7m1 and squares represent the volume of the DLP of b7m1.

The prostate of a castrated animal returns to pre-castrate size, and indeed larger, following androgen supplementation. Two weeks following castration, mice were given daily DHT injections and the prostates repeatedly imaged. Figure 5 shows re-growth over a two week period (panels A-F). The dorsal-lateral lobes (panels A'-F', black outlining) return to pre-castration volume by 9 days of treatment and then growth arrests (Figure 5G, squares). In contrast, the ventral lobe (white outline) returns to pre-castration volume by 9 days, but then increases further for the duration of the treatment (Figure 5G, circles).

## Discussion

We applied MR imaging to precisely quantitate the volume of the mouse prostate *in vivo *over multiple imaging sessions to follow regression and re-growth. This method should also be useful to follow tumor development and drug treatment effects in prostate and other organs. We have optimized the MRI technology protocol in a variety of ways to significantly improve on previous applications of MRI to prostate imaging in the mouse. Specifically, we refined the anesthesia administration protocol to allow imaging of a mouse for 3.5 hours. Extended imaging time is necessary to produce the highest quality images, by providing sufficient time to position the prostate at the focal point of the field, shim the magnetic field, and capture 8 averages of the image, enhancing image quality by improving the signal to noise ratio. We used a 256 × 256 matrix, a small (30 mm^2^) FOV, and very thin (200 μm) non-interleaved slices to obtain the smallest voxel size reported for mouse prostate imaging. Our protocol precisely determined the volume of the normal prostate from a single MRI session, as evidenced by the observation that volumes determined from additional imaging sessions did not vary significantly. Although developed independently, our method yields prostate volumes similar to those reported in MRI studies by Fricke, et al. [12], while our two-fold smaller voxel size due to a smaller coil and longer imaging times served to maximize the resolution and allow more precise volume determination. In rats, the wet weight of the ventral lobe is reduced 75% within 10 days of castration, while the dorsal and lateral lobes are only reduced 50% [14]. In this study, MRI revealed a 75% castration-induced volume loss for the whole prostate (Figure 2). Further, use of CHESS fat suppression to differentiate the lobes reveals 72% VP reduction and 39% DLP reduction (Figure 4). While direct comparison of the MR images to histological images at timed intervals following manipulation are a "gold standard" for accurate determination of prostate volume, using CHESS to optimize differentiation of prostate from surrounding tissue allows confidence in determining the organ boundaries used in prostate volume determination. Using MR to produce images of the same animal over time increases precision of the volume measurements due to elimination of inter-animal variation. Only five animals were needed to produce reliable data in the regression and re-growth studies, whereas a minimum of 4 animals/time point at 10 time points, 40 animals total, would be required to produce similar data histologically.

Mice deficient in genes regulating apoptosis have helped identify regulators of cancer development and progression in the rodent prostate [4]. Similarly, understanding the genetic basis of androgen withdrawal induced regression provides insight into androgen-independent progression of prostate cancer. Rat prostate lobes are large and can therefore be much more readily physically dissected from surrounding tissues. Regression can be monitored by sacrificing the rats, recovering the prostate and weighing individual lobes. Physical separation also allows measurement of protein content, DNA content, and nucleosomal fragmentation due to apoptosis. Two weeks following castration, the DLP mass declines 50% and VP declines 75% [14]. Unfortunately, rats are not yet a genetically tractable system and measuring apoptosis and regression in mouse prostate is technically challenging.

Since physical isolation of the lobes of mouse prostate is much more difficult than the rat [15], most investigators forego dissection and measuring regression and instead use histological techniques to measure apoptosis in the prostate. Pyknotic or TUNEL-labeled cells increase in the rat prostate following castration, but differences between castrated and normal are small (5 versus 30 per 100 acini) [16] such that many sections must be examined for accurate assessment. Moreover, pyknotic and TUNEL-stained cells are transient [17]. For these reasons, studies examining castration-induced regression of the mouse prostate have yielded contradictory results for some important signaling pathways, particularly Fas. To measure the effects of Fas deletion on androgen withdrawal induced regression of the prostate, both the organ size and rate of apoptosis have been measured in Fas-deficient (lpr) mice. Two studies sacrificed groups of animals at intervals following castration, microdissected and weighed the prostates. Since accurate microdissection of an interdigitated 20 mm^3 ^organ is technically challenging, it is perhaps not surprising that these groups reported contradictory results [18,19]. Two reports also measured the incidence of apoptotic cells by either histological characteristics [19] or TUNEL staining [20] in castrated Fas deficient mice and report no difference between deficient and normal mice. However, small differences in apoptotic or proliferation rate accumulate over time to yield measurable changes in prostate volume [3]. Thus, use of the MR imaging techniques described in this report, which integrate these small changes in the same animal, might provide a more accurate method to revisit the role of Fas in androgen withdrawal induced apoptosis and the resulting prostate regression.

## Conclusion

We have optimized very high resolution imaging of the mouse prostate in living animals. Our examination of the castration induced regression of the normal mouse prostate demonstrate that we can track the reduction in total prostate volume (Figure 2) and that using CHESS to suppress fat signal, we were able to further discriminate the ventral from the dorsal and lateral lobes of the prostate, and monitor volume changes in each individually (Figure 4). This additional level of anatomic discrimination should be useful in monitoring lobe-specific tumor growth in mice. We were also able to monitor prostate re-growth following administration of exogenous androgens (Figure 5). This MRI technique is sufficiently sensitive to detect even small changes in the size of the mouse prostate since monitoring of one animal repeatedly over time is more accurate than measuring the mean of a group of animals sacrificed at intervals after castration or other treatment. An added benefit is to dramatically reduce the number of animals needed to measure the effect of any given manipulation.

## Competing interests

The author(s) declare that they have no competing interests.

## Authors' contributions

KLN designed the studies, carried out the animal preparations, analyzed the imaging data and drafted the manuscript. HL carried out the MRI, analyzed the data, and designed the CHESS sequences. MH carried out the MRI, designed the T2 sequences and optimized the imaging analysis. LTM participated in the CHESS design, designed the coil, and helped to draft the manuscript. ON coordinated the study and participated in its design. JJK conceived of the study, and participated in its design and helped to draft the manuscript. All authors read and approved the final manuscript.

## Pre-publication history

The pre-publication history for this paper can be accessed here:


